# Insights into the Hydrocarbon Tolerance of Two *Devosia* Isolates, *D. chinhatensis* Strain IPL18^T^ and *D. geojensis* Strain BD-c194^T^, via Whole-Genome Sequence Analysis

**DOI:** 10.1128/genomeA.00890-15

**Published:** 2015-08-06

**Authors:** Yousef I. Hassan, Dion Lepp, Xiu-Zhen Li, Ting Zhou

**Affiliations:** Guelph Food Research Centre, Agriculture and Agri-Food Canada, Guelph, Ontario, Canada

## Abstract

Hexachlorocyclohexane (HCH) was among the most commonly used pesticides after the Second World War. The extensive use of this hydrocarbon for almost six decades has created a contamination problem on a global scale, and bioremediation methods are being extensively explored. The reported ability of some *Devosia* species to grow in the presence of appreciable amounts of hydrocarbons (2,000 mg/kg of contaminated soil) is attracting closer attention. Here, we report the *de novo* genome assembly of two hydrocarbon-tolerating *Devosia* isolates, *D. chinhatensis* strain IPL18^T^ and *D. geojensis* strain BD-c194^T^, as a first step toward understanding the metabolic pathways involved in their environmental adaptation and tolerance toward hydrocarbons.

## GENOME ANNOUNCEMENT

Hexachlorocyclohexane (HCH), a cyclic saturated chlorinated hydrocarbon insecticide, was among the most commonly used pesticides after the Second World War (1). The extensive use has created a contamination problem on a global scale, and bioremediation methods are being explored (2, 3). The ability of some *Devosia* species to grow in the presence of appreciable amounts of hydrocarbons (2,000 mg/kg of contaminated soil) is attracting attention within this context (4). Two strains, *Devosia chinhatensis* strain IPL18^T^ and *Devosia geojensis* strain BD-c194^T^, are of special interest. The first isolate emerged from an HCH dump site adjoining an India Pesticide Limited plant near Lucknow, India, and was identified by Kumar et al. (5); the other, *D. geojensis*, was characterized by Ryu and coworkers (4) from a diesel-contaminated soil sample collected from a gas (petrol) station in South Korea. Here, we report the *de novo* genome assembly of these two hydrocarbon-tolerating isolates as a first step toward understanding the metabolic pathways involved in their environmental adaptation and tolerance toward hydrocarbons.

*D. chinhatensis* IPL18^T^ (DSM24953) and *D. geojensis* BD-c194^T^ (DSM19414) were both obtained from DSMZ (Braunschweig, Germany) and reactivated, according to the provider’s recommendations. Overnight cultures were collected, and pellets were used to prepare the genomic DNA using the Puregene yeast/bacteria kit B (Qiagen, Toronto, Canada). Purified DNA was assessed using NanoDrop (Thermo Fisher Scientific, Waltham, MA) for quantity and quality parameters. Sequencing libraries were generated using the Nextera XT kit (Illumina, San Diego, CA), and 300-bp paired-end sequencing was performed with a MiSeq benchtop sequencer using a 600-cycle version 3 kit (Illumina). Following demultiplexing and quality filtering by the MiSeq Control Software version 2.5.0.5, human and *phiX*-contaminating reads were removed by mapping the filtered reads against the respective genomes using BBMap version 34.65 (http://sourceforge.net/projects/bbmap/). The average lengths of the final reads were 284 bp for *D. chinhatensis* and 266 bp for *D. geojensis*. The reads were assessed for quality with FastQC version 0.10.1 (Babraham Bioinformatics, Cambridge, United Kingdom) and assembled into contigs with the SPAdes assembler version 3.0.0 (6). The assembly quality was assessed with Quast version 2.3 (7).

The above-mentioned efforts generated two genomes that were submitted to the automatic annotation pipeline developed by NCBI. The resulting features of the *D. chinhatensis* strain IPL18^T^ genome were: a genome size of 3,497,719 bp, 98 contigs, an *N*_50_ of 2,139,066 bp, 255× genome coverage, 62.4% G+C content, 3,376 coding genes, 213 pseudogenes, 7 rRNAs, 47 tRNAs, and 3,108 proteins. Similarly, *D. geojensis* strain BD-c194^T^ yielded a genome size of 4,465,063 bp, 207 contigs, an *N*_50_ of 57,874 bp, 51× genome coverage, 4,314 coding genes, 269 pseudogenes, 65.9% G+C content, 3 rRNAs, 45 tRNAs, and 3,996 proteins.

### Nucleotide sequence accession numbers.

The following DDBJ/EMBL/GenBank accession numbers were obtained for the reported genomes: *D. geojensis* BD-c194 (JZEX00000000) and *D. chinhatensis* IPL18 (JZEY00000000). Both isolates are available from DSMZ (Braunschweig, Germany) under the accession numbers DSM24953 and DSM19414, respectively.
